# Bridging the phenotypic gap: Real-time assessment of mitochondrial function and metabolism of the nematode *Caenorhabditis elegans*

**DOI:** 10.1186/1472-6793-8-7

**Published:** 2008-04-02

**Authors:** Cristina Lagido, Jonathan Pettitt, Aileen Flett, L Anne Glover

**Affiliations:** 1 Institute of Medical Sciences, School of Medical Sciences, University of Aberdeen, Aberdeen, UK

## Abstract

**Background:**

The ATP levels of an organism are an important physiological parameter that is affected by genetic make up, ageing, stress and disease.

**Results:**

We have generated luminescent *C. elegans *through ubiquitous, constitutive expression of firefly luciferase, widely used for *in vitro *ATP determination. We hypothesise that whole animal luminescence reflects its intracellular ATP levels *in vivo*. To test this, we characterised the bioluminescence response of *C. elegans *during sublethal exposure to, and recovery from azide, a treatment that inhibits mitochondrial respiration reversibly, and causes ATP depletion. Consistent with our expectations, *in vivo *luminescence decreased with increasing sublethal azide levels, and recovered fully when worms were removed from azide. Firefly luciferase expression levels, stability and activity did not influence the final luminescence. Bioluminescence also reflected the lowered activity of the electron transport chain achieved with RNA interference (RNAi) of genes encoding respiratory chain components.

**Conclusion:**

Results indicated that *C. elegans *luminescence reports on ATP levels in real-time. For the first time, we are able to directly assess the metabolism of a whole, living, multicellular organism by determination of the relative ATP levels. This will enable genetic analysis based on a readily quantifiable metabolic phenotype and will provide novel insights into mechanisms of fitness and disease that are likely to be of relevance for other organisms, as well as the worm.

## Background

The physiology of model organisms is often less well understood than their genetics (phenotypic gap), but is essential to the understanding of gene function. We argue that the ATP levels of an organism are a relevant physiological parameter that is amenable for phenotypic analysis. Perturbation of an organism's ATP levels is an early indication of metabolic and physiological effects of disease, genetic make-up and stress. For example, one of the first manifestations of Alzheimer's is a decrease in energy metabolism in parts of the brain that correlates with a specific decrease in the activity of a key mitochondrial electron transport chain (ETC) enzyme, cytochrome c oxidase [1]. Deficiencies in energy metabolism are associated with other age-related metabolic and neurodegenerative diseases [2,3]. Impaired ATP production is a feature of Friedreich's ataxia [4] and Parkinson's disease (PD) [5,6]. A systemic defect in mitochondrial complex I is implicated in PD, and its pathological features have been reproduced by chronic exposure of mice to rotenone, a pesticide that specifically inhibits complex I [6].

The "steady state" ATP levels of an organism also reflect genetic factors. Knockdown of ETC genes in *C. elegans *resulted in decreased *in vitro *ATP levels [7], and deletion of genes in the insulin signalling pathway resulted in higher ATP levels [8,9]. In addition, there is an environmental influence, with exposure to stress, such as heat-shock [10], metabolic and oxidative stress [11-13], anoxia [14], and starvation [11,15,16] shown to decrease ATP levels and increase the AMP: ATP ratio.

Stress induced changes in ATP concentrations are often rapid and transient. Depending on the severity of stress, compensatory mechanisms aimed at restoring ATP levels are quickly activated [17]. Therefore stress responses are better studied in real-time. We describe here a novel sensor for real-time *in vivo *assessment of relative ATP levels in *C. elegans*.

We modified *C. elegans *to express firefly luciferase constitutively and ubiquitously throughout development. The enzyme is widely used *in vitro *to report on ATP levels: it catalyses the oxidation of luciferin in a reaction that consumes ATP and generates light and AMP [18]. Because *C. elegans *is transparent the *luc*-marked strains emit light when provided with exogenous luciferin. We have previously demonstrated a link between light levels and the worm's health upon exposure to environmental stress [19]. In this study, we generated highly luminescent strains of *C. elegans *that contained a luciferase gene, *luc+*, fused to the green fluorescent protein (GFP) gene, and specifically addressed the hypothesis that whole animal luminescence reflects its intracellular ATP levels *in vivo*. To test this hypothesis we characterised the bioluminescence response of *C. elegans *during sublethal exposure to, and recovery from sodium azide (NaN_3_), a treatment that inhibits mitochondrial respiration reversibly, and causes ATP depletion [3]. Consistent with our hypothesis, *in vivo *luminescence decreased with increasing sublethal azide concentration, and recovered fully when azide was removed. We then targeted mitochondrial respiratory chain components by RNAi, and have shown a decrease in luminescence that was consistent with the drop in *in vitro *ATP reported by Dillin *et al*. [7].

Although previously achieved at the single cell level [20,21], this is the first report to show that luminescence can be used for assessment of ATP in a living multicellular organism. This will enable genetic analysis based on a readily quantifiable metabolic phenotype and has the potential to further our understanding of the many genetic pathways involved in diverse aspects of *C. elegans *physiology, such as metabolism, ageing, disease and stress response. Since many of the *C. elegans *genes are well conserved [22], the significance of these findings will extend well beyond this organism.

## Results

### Strain characterisation and effect of sodium azide (NaN_3_) exposure

**Figure 1:**
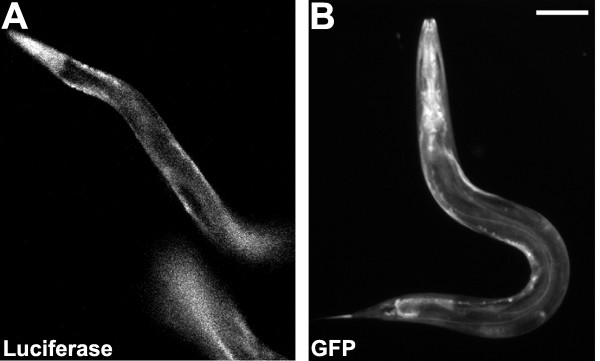
**Bioluminescent *C. elegans *expressing the LUC+::GFP**. **A**. Strain with luminescence array *feIs5 *in a *glp-4*(*bn2*) genetic background, displaying widespread *in vivo *luminescence. Image captured in complete darkness with 10 sec integration, after adding luminescence buffer plus 200 μM levamisole. Scale bar represents 100 μm. **B**. Image of a *feIs5 *worm shows that GFP expression is strongest in the pharynx and the tail areas. Widespread green fluorescence is shown by the *glp-4(bn2)*; *feIs5 *strain.

We have generated two independently integrated luminescent strains, PE255 *(feIs5) *and PE254 *(feIs4)*. The data we show were obtained with *feIs5*. Ubiquitous cytoplasmic expression of LUC+::GFP is shown by luminescent strains throughout development (Figure 1). Expression is strongest in the pharynx region and the tail area. Luminescence was detectable during late embryogenesis, all subsequent larval stages and in the adult (data not shown). Addition of luciferin was required for luminescence: the background signal prior to luciferin addition was negligible. Luminescence was enhanced 2.5 times through the addition of 1% DMSO and 0.05% triton-X (data not shown), provided to increase the permeability of the cuticle to luciferin [19].

**Figure 2:**
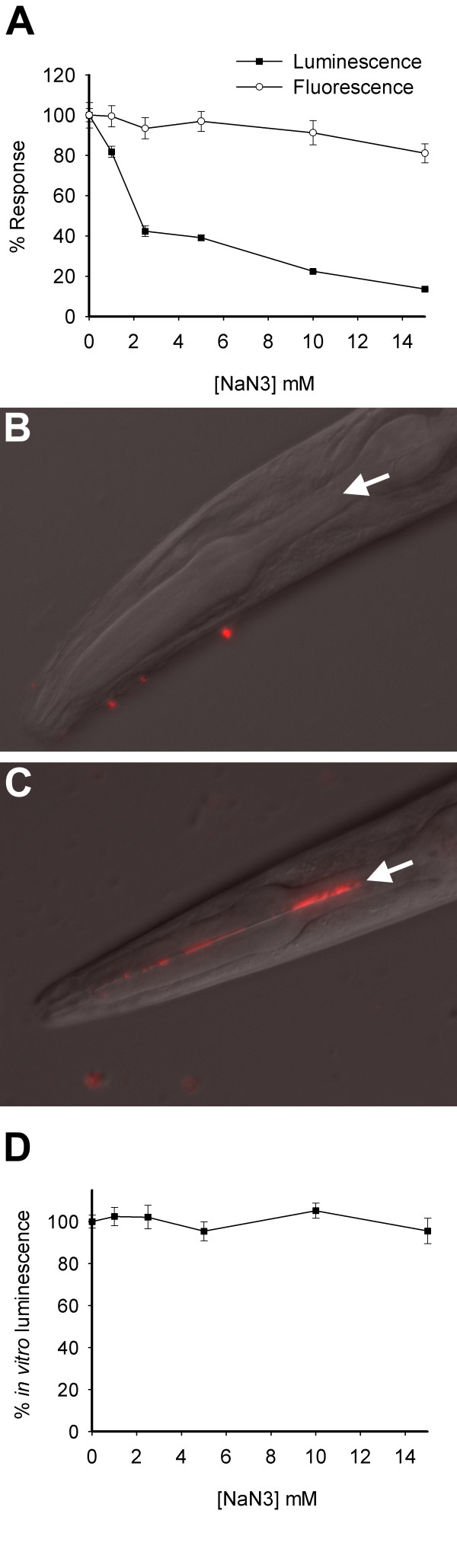
**Exposure of PE255 worms to the respiratory inhibitor sodium azide (NaN_3_) for 30 min**. **A**. Luminescence (*in vivo*) and fluorescence as a % of controls not exposed to NaN_3_. Luminescence decreased markedly with increasing concentration of NaN_3_, whereas survival remained 100% throughout (not shown). Fluorescence intensity tracked the GFP module of LUC+::GFP and was indicative of expression levels of the fused protein. It remained fairly constant throughout, except for 15 mM NaN_3 _where a small decrease was observed. The drop in luminescence was consistent with decreased ATP levels caused by NaN_3_. Synchronised L4 larval stage worms (from 45 h post hatch liquid cultures) were used. Error bars show the SEM. The pooled number of worms was 1200 (± 81) per tested condition. **B**. As a result of not feeding, a nematode treated with 1 mM azide for 30 min does not show red fluorescing microspheres in its pharynx. **C**. Red microspheres accumulate in the pharynx of control nematodes exposed to 0 mM azide. **D**. Luciferase activity (measured by *in vitro *luminescence) in lysates of worms exposed to NaN_3_, expressed as % of control values and normalised to protein content. There were no significant changes in luciferase activity with increasing NaN_3 _concentrations (Regression not significant; P = 0.623466), so that luciferase activity did not influence final *in vivo *luminescence. Error bars show the standard error of the mean (SEM). The pooled number of worms was 4000 (± 169) per tested condition.

We tested the effect of azide on worm bioluminescence. Exposure to 1–15 mM NaN_3 _led to a pronounced decrease in luminescence, without lethality (Figure 2A). All the tested concentrations of NaN_3 _stopped the pharynx activity as observed microscopically using a fluorescent microsphere assay. Microspheres were found only in the pharynx and intestinal tract of control worms not exposed to azide (Figure 2B–C). At concentrations above 1 mM NaN_3 _the worms were paralysed but alive, as assessed by their ability to regain movement within 4 h of removal of azide. Survival was 100% in each of the experimental conditions, proving that the drop in observed luminescence was not a result of lethality. To rule out the possibility that NaN_3 _affected LUC+::GFP expression, we measured the fluorescence intensity of worms exposed to NaN_3_. Up to 10 mM NaN_3_ there was little change in the intensity of fluorescence which reported on the GFP module of LUC+::GFP. In contrast, bioluminescence of worms exposed to the same concentrations of azide was depressed, falling to approximately 20% of its maximum values in worms exposed to 10 mM NaN_3_.

### *In vitro *determination of luciferase levels

To control for potential effects of NaN_3 _on luciferase protein levels, or its function, luciferase activity was measured in lysates of worms that were exposed to azide under the same conditions as for *in vivo *experiments (Figure 2D). A commercial kit that provided saturating levels of exogenous ATP and luciferin was used for this purpose. Luciferase activity was normalised to protein content of sample. No significant trend was observed between luciferase activity and NaN_3 _concentration (regression coefficient not significantly different from zero; P = 0.623466), therefore, under our experimental conditions the level and activity of luciferase did not account for the reduced light output in response to NaN_3 _(Figure 2D).

### Recovery from 30 min exposure to azide

**Figure 3:**
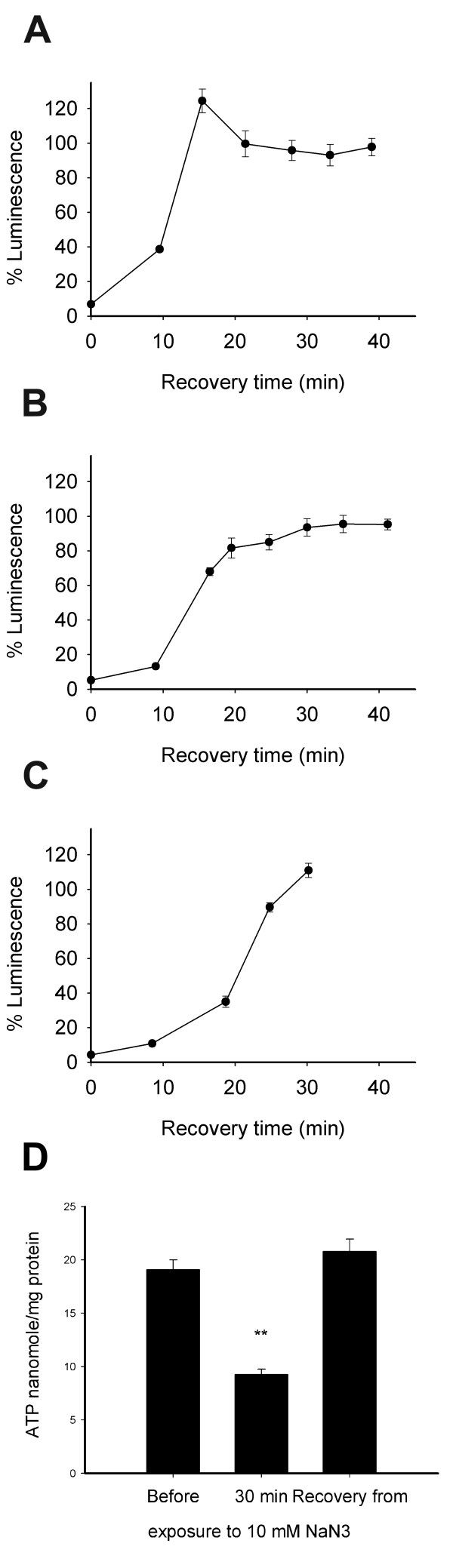
**Recovery from 30 min exposure to 10 mM sodium azide (NaN_3_)**. **A**. L3 stage worms (PE255), **B**. Young adults (PE255), **C**. Gravid adults (PE255). Luminescence (*in vivo*) increased rapidly from the time NaN_3 _was removed and reached the level of worms exposed to 0 mM NaN_3 _within 30 min. This fast recovery is consistent with recovery in ATP production, following removal of the respiratory inhibitor NaN_3_. Luminescence was expressed as a % of controls exposed to 0 mM NaN_3_. At each time point, the luminescence of independent samples was read following 3 min incubation with luciferin. Error bars show the SEM. The pooled number of worms was 2400 (± 198) per time point. **D**. *In vitro *ATP levels of N2 worms before, after 30 min exposure to 10 mM NaN_3_, and after 30 min recovery from 30 min exposure to 10 mM NaN_3_. Azide caused a significant (**, ANOVA, P < 0.001) reduction in the levels of *in vitro *ATP normalised to protein content. These levels returned to the levels of worms exposed to 0 mM NaN_3 _within 30 min of washing worms from the chemical (ANOVA, P > 0.05). Error bars show the SEM. The pooled number of worms was 4000 (± 429) per tested condition.

One characteristic of NaN_3 _is that its effects on ATP levels are reversible. To further establish the link between ATP levels and luminescence we have tested for recovery of synchronised L3 worms, young adults and gravid adults from exposure to 10 mM NaN_3_. Luminescence was measured as quickly as possible over time, after washing worms from NaN_3 _(Figure 3A–C) and following 3 min incubation with luciferin in each case. Rising luminescence was observed when worms were removed from azide, with full recovery within 30 min, as established by comparison with controls not exposed to NaN_3_. Younger worms (L3) recovered faster than older worms (gravid adults), possibly due to their smaller size and faster diffusion of azide from the nematode's tissues. In parallel, the *in vitro *ATP levels of wild-type N2 worms were measured prior to, after 30 min treatment, and following recovery from 10 mM NaN_3 _(Figure 3D). ATP levels, normalised to protein, were significantly reduced by azide (ANOVA, P < 0.001), but after 30 min recovery were restored to the levels measured prior to azide treatment (ANOVA, P > 0.05). Thus, the recovery of luminescence was consistent with the *in vitro *ATP results, showing a return to normal ATP levels once NaN_3 _was removed.

### RNAi knockdown of respiratory chain genes

**Figure 4:**
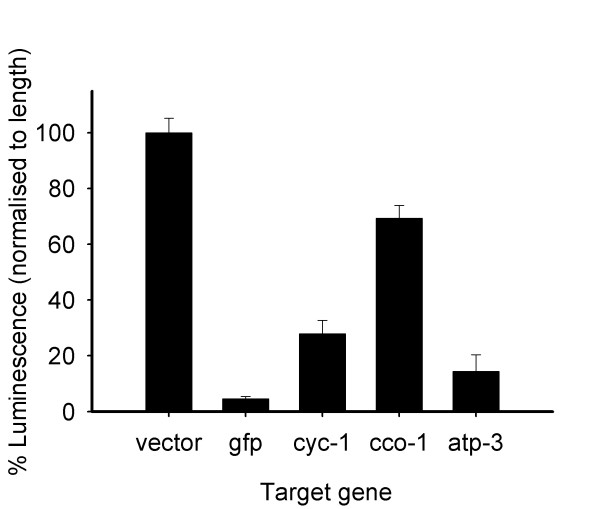
**Bioluminescence response following RNAi of respiratory chain genes**. Decrease in Luminescence was consistent with reported changes in *in vitro *ATP levels. Animals [*glp-4*(*bn2*); *feIs5*] were exposed to dsRNA from the L1 larval stage, for 4 days, at 25°C. Luminescence was normalised to average length of worms. Differences in intensity of fluorescence were only evident when *gfp *was targeted by RNAi. Luciferase expression is also lowered with the *gfp *RNAi clone, as the *luc*+ and *gfp *genes are fused. Error bars show the SEM. The pooled number of worms was 480 (± 26) per tested condition.

To deplete ATP levels without addition of exogenous chemicals, components of the mitochondrial respiratory chain were targeted by RNAi, and the outcome was assessed by luminescence. RNAi for respiratory chain genes caused a decrease in both worm size and luminescence in relation to controls (empty RNAi vector), therefore to control for the reduced size luminescence was normalised to length of worms (Figure 4). The relative body proportions were maintained by nematodes that showed reduced size and thus length was a suitable descriptor of body size. Knocking down *gfp *reduced fluorescence and luminescence dramatically whilst not affecting size of worms. This was indicative of decreased expression of LUC+::GFP and was the only instance where the intensity of fluorescence was decreased, as observed microscopically. We found that RNAi for: *cyc-1*, a cytochrome c reductase (complex III) gene, reduced luminescence to 28% of maximum levels observed for empty vector control; *cco-1*, a gene encoding a subunit of cytochrome c oxidase (complex IV), reduced luminescence to 69%; and *atp-3*, encoding a mitochondrial ATP synthase (complex V) gene, reduced luminescence to 14%. Thus, we have shown that reducing expression of respiratory chain genes has an effect on bioluminescence that is consistent with reported changes in ATP levels under similar conditions.

## Discussion

In *C. elegans*, assessment of ATP levels has until now been carried out *in vitro *[7-9,23], a method which is accurate but implies the destruction of worms, limits the scalability of the analysis and cannot be carried out in real-time. We report on the construction of a luminescent transgenic *C. elegans *strain and have tested the hypothesis that light emitted by living *C. elegans *is a reflection of its ATP pools. We used two approaches: i) exposure to and recovery from sodium azide, a specific mitochondrial inhibitor, and ii) RNAi towards genes that are essential for mitochondrial function. Azide inhibits complex IV of the mitochondrial respiratory chain by binding reversibly to cytochrome c oxidase [24], this arrests the flow of electrons and leads to a decrease in ATP synthesis. Because the inhibition is reversible azide has been widely used as a *C. elegans *anaesthetic [25]. Changes in bioluminescence upon exposure to, and recovery from, sublethal concentrations of azide were consistent with reversible ATP depletion caused by azide. Recovery in bioluminescence occurs within 30 min of removing worms from azide. Similarly, recovery of ATP depletion resulting from anoxia occurs within 45 min of reversal of anoxia [14]. Luciferase expression and activity levels stayed constant in the azide experiments and therefore did not contribute to observed changes in bioluminescence.

The substrate luciferin has to be present intracellularly for light emission. It is not known how luciferin enters *C. elegans*. It is possible that it passes through the permeabilised cuticle. Alternatively, it may be ingested through the pharynx. If an active pharynx was a requirement for luciferin entry, then substrate availability could be a limiting factor for luminescence under experimental conditions that alter the pumping rate, such as toxin exposure [26]. However, in this work, although exposure to azide stopped pharynx activity, the worms were able to emit light when provided with the substrate, indicating that luciferin was able to cross the permeabilised cuticle. Luciferin has been deemed to be poorly taken up by cells [27], however, empirical data suggest that luciferin uptake occurs readily. Luciferin crossed the blood-brain barrier in mice easily, passing through endothelial cells that are amongst the least permeable cells in the mouse [28]. Studies in other organisms such as *Drosophila *[29], zebra fish [30] and *Arabidopsis *[31] also indicated that diffusion and permeability of luciferin did not limit bioluminescence. Furthermore, we have observed that luciferin was capable of crossing the shell of unhatched *C. elegans *embryos, considered poorly permeable to chemicals, resulting in bioluminescence. We have also captured images of widespread luminescence in the worm's tissues, contrary to what would be expected if luciferin was poorly taken up by cells.

Inhibition of respiratory chain components by RNAi provided a means of depleting ATP levels without exogenous chemicals. The response measured by bioluminescence is in agreement with *in vitro *data. *In vitro *ATP was reduced to 20–40% when *cyc-1 *or *atp-3 *were knocked down, and to 40–60% for *cco-1 *RNAi [7]. All experimental data were consistent with the hypothesis that *C. elegans *luminescence reflects its ATP pools. Additionally, the RNAi experiments illustrate bioluminescence as a phenotype that could be the basis for genetic analysis.

Wildtype firefly luciferase is targeted to peroxisomes [32] and has a 3 h half-life [27,33]. In this study, the nematodes were transformed with a modified firefly luciferase gene *luc*+ fused to *gfp*, which is not targeted to the peroxisomes. Luc+ is expressed in the cytosol and has a half-life of 10 h in human breast cancer cells [34]. The half-life of GFP is 26 h [35] and therefore should not contribute to instability of the fusion protein. The greater stability of Luc+ is an advantage for studies where ATP changes may be tracked over time, as opposed to the requirement for a short half-life when luciferase reports on gene expression.

One critical aspect that will affect the luminescence readings is the levels of firefly luciferase present, this may vary for example between different strains and developmental stages. Hence, the exact relationship between light output and ATP concentrations will depend on the experimental conditions, ruling out precise determination of ATP content. We propose this strain as a relative sensor of ATP levels which can be applied to interrogate mitochondrial function and metabolism of living worms in a non-destructive, real-time and scalable manner. This will have broad appeal as sublethal physiological parameters are often difficult to quantify, especially on a large scale. Perhaps the most exciting developments will be the identification of novel genes and pathways underlying physiological response, as well as a better understanding of classical pathways. The integrated *luc+:: gfp *fusion we described can be crossed into available strains carrying gene deletions or into new mutants generated by mutagenesis. Alternatively, any of the worm's genes can be targeted for inactivation by RNAi and bioluminescence will provide an easily quantifiable metabolic phenotype. The luciferase gene may also be placed under promoters that will drive its expression in specific tissues and allow for relative assessment of ATP levels in that tissue. Ballistic transformation methods will enable expression of the transgene in the germline where required. The transgenic strains described here offer a unique opportunity to explore the links between physiology and genetics of *C. elegans *and many other organisms with which it shares homologue genes, including humans [22].

## Conclusion

We have described the construction of highly luminescent strains of *C. elegans *and have shown that emitted light is a reflection of their ATP pools. Changes in bioluminescence upon exposure to, and recovery from, sublethal concentrations of a respiratory inhibitor were consistent with the levels of ATP determined *in vitro *for similar conditions. Luciferase activity levels remained stable throughout and consequently did not contribute to the measured changes in bioluminescence. We also presented evidence that luciferin crossed the permeabilised worm cuticle.

We used RNAi towards ETC components to reduce ETC activity and lower ATP synthesis. For the first time mitochondrial function of a whole multicellular organism was determined by *in vivo *luminescence, with light levels providing a sensitive measure of the ATP levels. Thus, luminescence proved an efficient means for detection of sublethal energy related RNAi phenotypes.

Deciphering gene function is an important challenge in the post-genomic era. Often genes conferring morphological traits are the easiest to study, whereas those involved in sublethal behaviour and physiology prove hardest. The ATP levels of an organism often provide an early manifestation of changes induced by stress and mitochondrial diseases. Our strains will enable rapid real-time assessment of relative ATP levels of a living model organism and will further the research of its biology.

## Methods

### Construction of plasmid pSLGCV

The backbone for pSLGCV was plasmid pPD95.79 (Firelab, Carnegie Institution for Science), which contains *gfp*. To this backbone we added sequentially the *sur-5 *promoter [36], PCR-amplified from plasmid pTG96 and then the luciferase gene PCR-amplified from vector pSP-*luc*+ (Promega). A 3.7 kb fragment upstream of the *sur-5 *gene containing the promoter, was amplified using primers 5'- ATAAGCTTGCATGCCTGCATTGC-3' and 5'-AGACACCCCGGGCTTTCTGAAAAC-3' (this introduces a *Sma *I site and removes the start codon for the *sur-5 *gene). The *sur-5 *promoter was inserted in the pPD95.79 backbone using appropriate restriction sites (*Sph *I and *Sma *I).

The *luc+ *gene was amplified using primers 5'-TCCCGGGAAGCTTTCCATGGAAGAC-3' and 5'-CTAGGGTACCACGGCGATCTTTCC-3'. *Sma *I and *Kpn *I were used to insert *luc+ *downstream of the *sur-5 *promoter and upstream of and in frame with *gfp*. The peroxisome tagging sequence is absent from *luc+ *and the NLS for *sur-5 *was excluded from pSLGCV, so that LUC+::GFP was expressed in the cytoplasm.

### Strain construction

Microinjection of the gonad syncytium was carried out as standard [37]. The plasmid pSLGCV was co-injected with the marker pRF4 at 250 ng/ml each. A transgenic line, *feEx44 [sur-5::luc+::gfp; rol-6(su1006)]*, was isolated and the array was integrated by EMS mutagenesis [38], followed by selection of F2 animals that gave rise to 100% roller progeny. We obtained two independently integrated lines, the first into the X chromosome and the second where the transgene appears to be in chromosome V. The first line was out-crossed 8 times with the wild-type N2 strain to generate PE255 (*feIs5*), and the second was out-crossed 10 times to generate PE254 (*feIs4)*. The data shown were obtained with *feIs5*, except where otherwise stated.

### Measurement of luminescence

Luminescence was measured in a Clarity microplate luminometer (Biotek) in the visible spectral range between 300 and 600 nm, (firefly luciferase typically emits at 550–570 nm). White microplates were used (Greiner) with approximately 100 worms per well (in 100 μl). An automated dispenser delivered 50 μl of luminescence buffer to each well, consisting of citrate phosphate buffer pH 6.5, 0.1 mM D-luciferin, 1% DMSO and 0.05 % triton-X (all final concentrations). Luminescence of the PE255 strain increases steeply in the first minute after adding luciferin, followed by a slower increase to its maximum levels observed within the second minute. The light levels remain fairly stable during the first 5 minutes, followed by a gradual decrease to 60–80% of the maximum luminescence values in the first half hour (Additional file 1). Luminescence was read for 1 sec, 5 min after adding luciferin, except for the azide recovery experiments, where incubation with luciferin was only 3 min. These incubation periods allowed for maximum luminescence to be reached. During incubation with luciferin plates were shaken at 160 rpm. Except where otherwise mentioned, all luminescence measurements were carried out *in vivo*.

### Measurement of fluorescence

Fluorescence was quantified in a FLUOstar OPTIMA microplate reader (BMG labtech) using 485 nm excitation filter and a 520 nm emission filter. Background measurements were subtracted from readings.

### Sodium azide (NaN_3_) exposure bioassays

Worm cultures were synchronised by bleaching and overnight hatching in M9 [39]. L1 stage nematodes were washed and incubated (20°C, 160 rpm) for 45 h in S medium supplemented with 15 g/L *E. coli *OP50, at a density of 20 to 30 worms per 10 μl. 50 μl of the worm culture was aliquoted to each well of 96-well plates, white for luminescence or black for fluorescence. 50 μl of sodium azide (SIGMA) was added per well to final concentrations of 0, 1, 2.5, 5, 10 and 15 mM. Each concentration was tested on 8 replicate wells. The plates were covered with air permeable seals (ABgene), and incubated at 20°C, 160 rpm, for 30 min prior to measurement of light output or fluorescence. To assess survival worms exposed to each NaN_3 _concentration were pooled and washed 3 times with S basal [39], supplemented with 0.01% Tween-20 to prevent worms from sticking to plasticware. Triplicate 10 μl samples were plated on to NGM plates [39] and viability assessed by motility within 4 h.

### Pharyngeal pumping assay

Worms were treated as described in NaN_3 _exposure bioassays. An adaptation of the method of Mörck *et al*. [40] was used for assessing pharyngeal pumping. Briefly, after exposure to the various azide concentrations, Fluoresbrite™ Polychromatic red microspheres, 0.5 μm diameter (Polyscience, Inc), suspended in luminescence buffer, were added to each well, to a final dilution of 50 times. After 10 min contact time, worms were washed with M9 plus 1 mM levamisole (600 × g, 2 min), transferred to 2% agarose (w/v in M9) pads and mounted for observation under fluorescent microscopy (Texas red filters). A minimum of 30 worms were scored for the presence of microspheres in their pharynx and gut, for each of the azide concentrations tested.

### Recovery from azide

Synchronised worms were exposed to 10 mM NaN_3 _for 30 min, in S complete supplemented with *E. coli *OP50. Luminescence was read just prior to washing worms from NaN_3_, and this reading was adopted as the zero time point for recovery (t = 0). Recovery was timed from removal of azide solution. Worms were then washed once, centrifuged (1 min, 600 × g) resuspended in S complete with 7.5 g/L *E. coli *OP50, and aliquoted to 96-well plates. Luminescence was measured at different time points, as quickly as possible, during the first half hour of recovery. At each time point, a different set of samples was read, so that the incubation time with luciferin was the same (3 min) each time. Each luminescence time point is the average of 24 replicate wells. Luminescence of worms not exposed to azide was taken as the guideline for full recovery.

### *In vitro *luciferase assay

Exposure to 10 mM azide was carried out as described above. Worms were then washed 3 times (1 min, 600 × g) in S basal, and approximately 1000 worms in 100 μl of S basal were frozen in liquid nitrogen and stored at -80°C until analysis. Four replicate samples for each azide concentration were thawed on ice and 200 mg glass beads (212–300 μm) and CCLR reagent were added (Promega). To obtain worm lysates samples were put through a Fastprep homogeniser 8 times (5000 rev/min, 30 s) with incubation on ice in between runs. Lysates were analysed using Luciferase Assay system (Promega), according to manufacturer's instructions. The *in vitro *luminescence data were normalised to protein content of samples.

### *In vitro *ATP determination

A luminometric method adapted from Braeckman *et al*. [23] (Braeckman pers. comm.) was used. Briefly, four replicate samples of washed worms were collected in 100 μl of S basal, quickly frozen in liquid nitrogen and stored at -80°C. Worms were broken up by disruption with glass beads (212–300 μm) in a Fastprep homogeniser after addition of 8 % (v/v) HClO_4_. Extracts were neutralised with 1.3 M KHCO_3_, and buffered with 1 M K phosphate buffer (pH 7.6) prior to measurement of ATP concentrations with the ATP bioluminescence CLS II kit (Roche), in a Clarity luminometer (Biotek). ATP concentrations were normalised to protein content of samples.

### Protein determination

BCA kit (Pierce) was used in all protein determinations according to manufacturer's instructions, with incubation at 60°C.

### RNAi Knockdown of respiratory chain genes

The Rivers *et al*. protocol for RNAi feeding in liquid culture was used [41]. Approximately 15 synchronised L1 nematodes were placed in each well of 96 -well microplates and cultivated with bacteria expressing dsRNA for respiratory chain genes (MRC GeneService clones), or control clones (GFP clone: pPD128.110; empty vector control: pPD129.36; both Firelab vectors), for 4 days at 25°C without shaking, prior to measurement of *in vivo *luminescence (as above) and length. To control for reduced body size, luminescence readings were normalised to average worm length. A microscope equipped with an ocular micrometer was used to measure worms after immobilisation with levamisole (200 μM). At least 30 measurements were taken for each experimental condition.

### Imaging of worms

A Photometrics CASCADE II 512 Air cooled (-70°C) CCD sensor and a Deltavision microscope were used to capture images of the *glp-4(bn2)*; *feIs5 *strain. Worms were picked into 5 μl of luminescence buffer plus 200 μM levamisole (to immobilise worms) and mounted in 2% agarose pads. Luminescence was captured with no incident light, in a dark chamber, with 10 sec integration, 18 min (± 2 min) after treatment with the luminescence buffer plus 200 μM levamisole. As a control, conditions for imaging luminescence were tested on worms that carry the plasmid PTG96 [36] and display expression of GFP on its own. No luminescence was captured for the control. For imaging fluorescence illumination through FITC filters was used with an exposure time of 0.2 seconds. A Zeiss Axioplan2 microscope with a HAMAMATSU ORCA-ER CA742–80 digital camera was used for additional photographs of worms, including those of the *feIs5 *strain and the pharyngeal pumping assay.

### Statistical analysis

Statistical analysis was performed with SPSS 15.0 and Microsoft Office Excel 2003. Least significant differences (LSD) [42] were calculated for statistically significant ANOVA results.

## List of abbreviations used

ATP, adenosine 5'-triphosphate; RNAi, RNA interference; AMP, adenosine 5'-monophosphate; ETC, electron transport chain; PD, Parkinson's disease; GFP, Green fluorescent protein; DMSO, dimethyl sulfoxide; NLS, nuclear localisation signal.

## Authors' contributions

CL conceived and designed the study, constructed strains, carried out experiments, analysed data and wrote the manuscript. JP and LAG conceived, oversaw the design and execution of the study and revised the manuscript. JP participated in the construction of strains. AF contributed significantly to experimental work and revised the manuscript. All authors read and approved the final manuscript.

## Supplementary Material

Additional file 1 **Time course of strain PE255 luminescence following addition of luciferin (at t = 0)**. Luminescence buffer, consisting of citrate phosphate buffer pH 6.5, 0.1 mM D-luciferin, 1% DMSO and 0.05 % triton-X (all final concentrations) or 0.1 mM D-luciferin (final concentration) in citrate phosphate buffer pH 6.5 (without DMSO or triton-X) was added to wells containing 15 unsynchronised gravid worms. Luminescence was measured in a Clarity luminometer using the KC4 programme. Luminescence increased rapidly after adding luciferin, reaching its maximum levels within the second min, but remaining relatively stable for the first 5 min, followed by a gradual decrease in luminescence. The presence of 1% DMSO and 0.05 % triton-X increases luminescence by 2 to 2.5 times. Click here for file
