# Supplementary figures and images for: Bridging the phenotypic gap: Real-time assessment of mitochondrial function and metabolism of the nematode Caenorhabditis elegans
Source: BMC Physiol. 2008 Apr 2;8:7. doi: 10.1186/1472-6793-8-7 (PMC2364618; doi:10.1186/1472-6793-8-7)

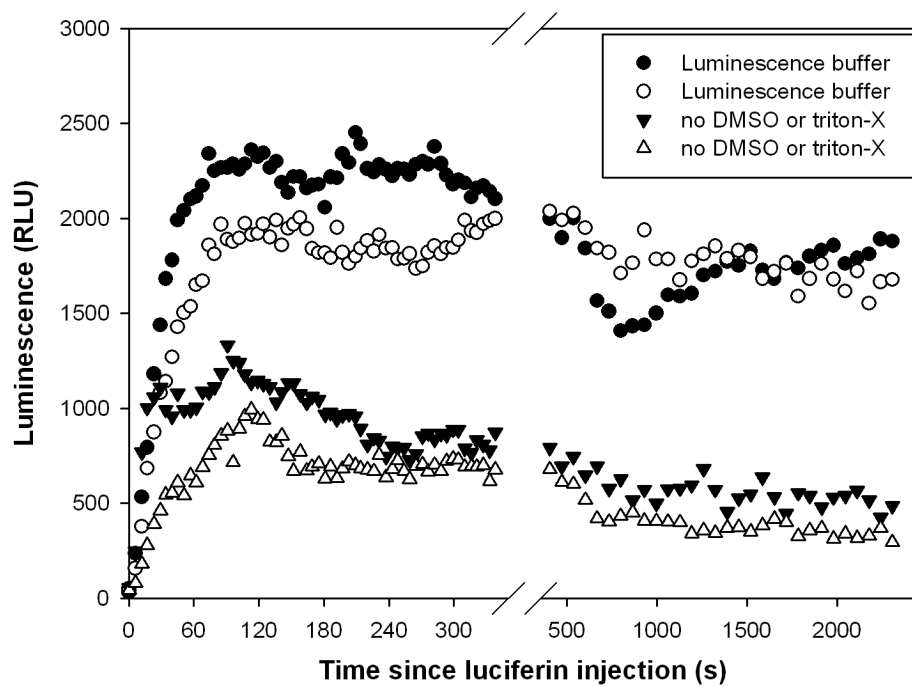

Supplement: Additional file 1 — Time course of strain PE255 luminescence following addition of luciferin (at t = 0). Luminescence buffer, consisting of citrate phosphate buffer pH 6.5, 0.1 mM D-luciferin, 1% DMSO and 0.05 % triton-X (all final concentrations) or 0.1 mM D-luciferin (final concentration) in citrate phosphate buffer pH 6.5 (without DMSO or triton-X) was added to wells containing 15 unsynchronised gravid worms. Luminescence was measured in a Clarity luminometer using the KC4 programme. Luminescence increased rapidly after adding luciferin, reaching its maximum levels within the second min, but remaining relatively stable for the first 5 min, followed by a gradual decrease in luminescence. The presence of 1% DMSO and 0.05 % triton-X increases luminescence by 2 to 2.5 times. [file 1472-6793-8-7-S1.pdf]
